# Motivators and barriers to vaccination of health professionals against seasonal influenza in primary healthcare

**DOI:** 10.1186/s12913-018-3659-8

**Published:** 2018-11-14

**Authors:** Davorina Petek, Kristina Kamnik-Jug

**Affiliations:** 1 Department of Family Medicine, Faculty of Medicine, University of Ljubljana, Poljanski nasip 58, 1000 Ljubljana, Slovenia; 2 Primary Health Care Centre Slovenj Gradec, Partizanska pot 16, 2380 Slovenj Gradec, Slovenia

**Keywords:** Influenza vaccination, Healthcare worker, Primary care, Risk groups

## Abstract

**Background:**

Over the last decade, the vaccination rates amongst the general population in Slovenia were declining. According to the World Health Organisation, the vaccination rates amongst healthcare workers are also low throughout Europe. The aim of this study was to evaluate vaccination rates for seasonal flu amongst healthcare workers on the primary care level in the Koroška region and to find motivators and barriers for vaccination.

**Methods:**

In a cross-sectional study, an anonymous questionnaire was distributed to all health centres, nursing homes and private contractors, who agreed to participate. Out of 334 distributed questionnaires, 250 (74.8%) were analysed. Bivariate and multivariable statistical analyses were conducted.

**Results:**

Only 12% of included health professionals were vaccinated in the 2014/15 season. The main motivators for vaccination coverage were: awareness of high risk of infection at the workplace, self-protection and protection of family members and co-workers. The main barriers for vaccination were doubt in the effectiveness of the vaccine, fear of side effects and the belief that health professionals are not at high risk of influenza infection. In the multivariable model, a positive association was found between the vaccination, older age and belief in the effectiveness against influenza, while a negative association was found between the nurses’ profession and vaccination.

**Conclusion:**

The trend of declining rates for seasonal influenza vaccination is continuing. Protection of the patients was not among the most important reasons for vaccination. This especially endangers clients of nursing homes. The recognized motivators, barriers and other factors that were important for vaccination coverage/hesitancy could be used for designing strategies and educational programmes for health professionals to improve the vaccination coverage rates. The strategy should include the specifics of health profession groups.

## Background

Influenza is an acute respiratory tract infection caused by influenza viruses. Seasonal influenza causes an overload of medical services on all levels of medical care and a high influx of patients admitted into hospital. In addition to over-burdened hospitals, these outbreaks also cause major healthcare system disorders, due to a lack of medical staff that suffer from influenza during these outbreaks [1, 2].

Vaccination is the most important public health measure for the prevention of seasonal and pandemic flu. Vaccination aims to reduce the population’s illness rate, the number of patients treated in hospitals and the mortality rate due to influenza complications. Vaccination of health professionals is also a way of maintaining full functionality of the healthcare system and protecting the patients during an epidemic or pandemic [1].

Health professionals are highly exposed to influenza in their workplace and can transmit the infection onto others. The World Health Organization (WHO) therefore classifies health professionals into the group of people for whom vaccination against influenza is recommended [3]. According to studies, approx. 20% of health professionals test positive for serologically confirmed influenza viruses during an epidemic. Infections are often asymptomatic, or - in 50% of the cases - subclinical. These individuals represent the potential source of infection at their workplace for patients and their co-workers [4].

Vaccination has been shown to reduce the number of serologically confirmed infections [1]. In view of this, vaccination is recommended as a preventive measure for self-protection and, consequently, an indirect protection of patients, co-workers, family members and others [4]. Preventing the infection of health professionals is also extremely important for the undisturbed functioning of the system, especially in case of major influenza outbreaks [5].

The evidence that vaccination of health professionals is effective for the protection of patients against influenza and influenza-like infections is relatively scarce [4, 6]. Some studies report a reduced mortality rate, fewer consultations with the family physician and fewer hospitalizations for nursing home residents at the time of an outbreak, if the staff had been vaccinated [3, 7].

The WHO and the National Institute of Public Health in Slovenia (NIJZ) encourage and promote vaccination of health professionals. Nevertheless, the vaccination coverage amongst Slovenian health professionals remains very low. Furthermore, there is a lack of records on the vaccination of employees in healthcare organizations [8].

Numerous studies that have examined health professionals’ views on vaccination have divided reasons for rejecting the vaccine into two major groups. The first group is the misrepresentation and misconception of influenza and its risks, the role of health professionals and the possibility that they infect their patients, and also the importance of vaccination, its effectiveness and safety. The second major group is a lack of easy access to free vaccine [9–12].

The law in Slovenia dictates the vaccination of health professionals who can be exposed to biological agents at their workplace or who can infect other people. Vaccination-related issues are controlled by the ‘Directive on the protection of workers from risk related to exposure to biological agents at work’. This Directive provides that employers must, according to their national law, offer their employees free vaccination on the basis of a risk assessment of exposure to biological agents against an effective vaccine [13, 14]. It follows that employers should cover the cost of influenza vaccinations and offer it to their employees free of charge, but the vaccination against influenza is not obligatory for health-workers.

Some of the healthcare organizations in other countries have decided to mandate the vaccination of health professionals against seasonal influenza [15]. Studies have confirmed that mandatory vaccination, where sanctions for non-compliance are not envisaged, does not have the same impact [8, 15]. Mandatory vaccinations for health professionals could be very effective, but at the same time they raise a number of issues on the freedom of choice. In comparison to mandatory vaccinations, the recommendation to get vaccinated is the milder approach towards achieving higher levels of vaccination coverage [8].

The aim of this study was to evaluate vaccination rates for seasonal flu amongst health professionals at the primary care level in Slovenia and to find motivators and barriers for the vaccination.

## Methods

### Sampling

In the cross-sectional study below, the data for analysis was gathered with an anonymous questionnaire for the 2014/15 vaccination season, from April to June 2015.

We invited all major healthcare providers in primary care in the region, among them primary healthcare centres with associated emergency services, nursing homes and private contractors. The list of health professionals was obtained via the National Institute for Public Health’s (NIJZ) freely accessible database of primary health professionals and covered the following positions: physicians, dentists, practice and registered nurses, community nurses, paramedics, nurse-carers, physiotherapists and occupational therapists.

The vaccination rate in 2014/15 was compared to the rate in 2013/14. We calculated the number of the vaccinated health workers in 2013/2014 from the database of the NIJZ (the number of all health workers [16] in the region and the number of vaccinated health workers in the region in 2013/14 [17].

### Questionnaire

The anonymous questionnaire made up of 27 questions and divided into 5 sections was designed based on both the review of specialised literature and the overall aim of the study. We have designed the questions on the basis of domestic and international literature [9–12] and national “grey” literature – specialist thesis that also involved qualitative research (semistructured interviews) with chronic patients and their attitudes towards vaccination against flu. The sections covered the demographic data of the participants, questions about health characteristics, knowledge and beliefs of influenza vaccination, and the reasons pro or against being vaccinated (multiple choice questions).

### Variables

The outcome variable was vaccination against influenza in the season 2014/15. Several explanatory variables about potential reasons for and against vaccination were tested for associations with the vaccination. The multivariable logistic regression model included demographic characteristics (gender, age over 50 years, occupation of health professional), health characteristic (self reported presence of chronic disease), free vaccination availability, being well informed about the influenza (self assessed knowledge), beliefs about exposure at the work place, safety and effectiveness of the vaccine..

### Data analysis

The data was processed with the IBM SPSS 22 (IBM Corp., Armonk, NY) software. The statistical analysis included descriptive statistics, which was presented by frequencies and percentages or by mean values ± standard deviations. Binary logistic regression using the standard entry method was apllied to determine the associations between the explanatory variables discussed and influenza vaccination. Crude and adjusted odds ratios with 95% confidence intervals were calculated. The explanatory variables included in the multivariable regression model were adjusted among each other. The explanatory variables included in the multivariable regression model were also tested for multicollinearity using variance inflation factor (VIF) [VIF = 1/(1-R^2^)]. R^2^ (Nagelkerke R^2^ for logistic regression) was obtained by regressing each explanatory variable on the remaining explanatory variables in the model. Statistical significance was set at *p* < 0.05.

## Results

### Sample description

Four hundred and eighty nine primary health professionals from the national list of healthcare professionals in the Koroška region were invited to participate in the study. Three hundred and thirty four healthcare professionals agreed to participate. The questionnaire, delivered in paper form, was completed by 263 health professionals (53.7%), while 13 were excluded from the analysis due to incomplete data. The final sample size therefore amounted to 250 health professionals (51.1% of the eligible sample and 74.8% of the distributed questionnaires). The basic characteristics of participants are listed in Table 1.

**Table 1:** A description of the sample of participating health professionals

Health professionals/institutions	Included *n* = 250 (%)	*N* (number of vaccinated) %
Sex		
Male	62 (24.8)	8 (12.9)
Female	188 (75.5)	22 (11.7)
Occupation		
Physician	44 (17.6)	13 (29.5)
Nurses	129 (51.6)	12 (9.3)
Paramedic	17 (6.8)	0 (0)
Dentist	14 (5.6)	4 (28.6)
Other health professionals	46 (18.4)	1 (2.2)
Type of healthcare institution		
Health centre	124 (49.6)	23 (18.5)
Nursing home	81 (32.4)	2 (2.4)
Private contractor	21 (8.4)	5 (23.8)
Emergency centre	24 (9.6)	0 (0)

The mean age of health professionals was 41.5 ± 12.2 in the range of 19 to 74 years. Among physicians, family and general practitioners were dominant (37 out of 44 or 84.1%); 10 out of 37 general practitioners (27.0%) were vaccinated.

In the 2014/15 vaccination season, 30 people or 12.0% of health professionals in our sample were vaccinated which is 5% less than the year before. As the data on vaccination of primary care workers was not available, we could compare the vaccination rate in 2013/14 of all health workers in the region from the national database of vaccination. Out of 1113 health workers, 192 people or 17.3% of health professionals were vaccinated in the 2013/14 vaccination season [17]. The difference was statistically significant (chi^2^ = 4.121; *p* = 0.042).

The reasons for vaccination are shown in Table 2. The two most important reasons for vaccination, expressed by health professionals, were belonging to a risk occupational group (83.3%) and self-protection against influenza (70.0%).

**Table 2:** Reasons FOR influenza vaccination in the 2014/15 season

Reasons for vaccination	*n* = 30	%
As a health professional, I belong to the risk group for infection	25	83.3
Self-protection against influenza	21	70.0
Protection of family members, co-workers	18	60.0
Protection of patients	14	46.7
My employer offers free vaccination against seasonal influenza	11	36.7
Easy access to vaccine or vaccination	11	36.7
Age over 50 years	8	26.7
I have a chronic illness	2	6.7

In the 2014/15 season, 220 (88.0%) health professionals did not vaccinate against seasonal influenza. The reasons for the decision against vaccination are presented in Table 3. The two most important reasons against vaccination were not being directly occupationally exposed (37.3%) and doubting the effectiveness of the vaccine (37.3%).

**Table 3:** Reasons AGAINST influenza vaccination in the 2014/15 season

Reasons against vaccination	*n* = 220	%
I do not belong to the influenza infection risk group	83	37.7
I have doubts in the effectiveness of the vaccine	82	37.3
Because of the adverse effects of the vaccine	67	30.5
Lack of time	15	6.8
I do not have sufficient information on the benefits of the vaccination and the consequences of the disease	8	3.6
Financial reasons	6	2.7
I am allergic to one of the components of the vaccine	4	1.8
Poor vaccine availability	0	0.0

### Associations of demographic data, health status, occupational exposure and beliefs about influenza vaccination in the 2014/15 season

Table 4 presents the results of logistic regression of the factors associated with influenza vaccination. Age above 50 years (OR = 3.73, 95%CI = 1.27–10.97, *p* = 0.017) and belief that the vaccine is effective in prevention of influenza (OR = 12.38, 95%CI = 4.06 –38.30, *p* < 0.001) were positively associated with influenza vaccination. Nurse occupation (OR = 0.24, 95%CI = 0.08–0.75, *p* = 0.014) was negatively associated with influenza vaccination in comparison to physicians. Included variables in the multiple logistic regression model explained 51.6% of the variance of the dependent variable (Nagelkerke R^2^ = 0.516). Regarding multicollinearity the maximum VIF among explanatory variables was 1.57, which was substantially below the rule-of-thumb cutoff of 4 [18], so there were no issues with multicollinearity.

**Table 4:** Associations of demographic data, health status, occupational exposure and beliefs about vaccination to influenza vaccination in the 2014/15 season

	Without *n* = 220 (%)	Vaccinated *n* = 30 (%)	cOR (95% CI)	p	aOR (95% CI)	P
Sex						
Male	54 (24.5)	8 (26.7)	1.00 (basis)		1.00 (basis)	
Female	166 (75.5)	22 (73.3)	0.90 (0.38–2.13)	0.801	0.60 (0.17–2.13)	0.431
Age above 50 years	49 (22.3)	17 (56.7)	4.56 (2.07–10.04)	< 0.001	3.73 (1.27–10.97)	0.017
Occupation						
Physicians	31 (14.1)	13 (43.3)	1.00 (basis)		1.00 (basis)	
Nurses	117 (53.2)	12 (40.0)	0.25 (0.10–0.59)	0.002	0.24 (0.08–0.75)	0.014
Paramedics	17 (7.7)	0 (0.0)	0.65 (0.35–1.61)	0.413	0.36 (0.08–1.65)	0.332
Dentists	10 (4.5)	4 (13.3)	0.95 (0.25–3.60)	0.944	2.28 (0.28–18.54)	0.442
Other health professionals	45 (20.5)	1 (3.3)	0.53 (0.01–0.43)	0.006	0.20 (0.02–2.04)	0.175
Presence of chronic illness						
no	196 (89.1)	22 (73.3)	1.00 (basis)		1.00 (basis)	
yes	24 (10.9)	8 (26.7)	2.97 (1.19–7.40)	0.020	2.74 (0.68–10.99)	0.154
Institution offers free vaccination						
no	95 (43.2)	4 (13.3)	1.00 (basis)		1.00 (basis)	
yes	125 (56.8)	26 (86.7)	4.94 (1.67–14.63)	0.004	2.29 (0.62–8.56)	0.216
Well informed of the nature of the disease						
no	67 (30.5)	3 (10.0)	1.00 (basis)		1.00 (basis)	
yes	153 (69.5)	27 (90.0)	3.94 (1.16–13.44)	0.028	2.36 (0.48–11.73)	0.293
Vaccination is effective in prevention of influenza						
not agree	155 (70.5)	5 (16.7)	1.00 (basis)		1.00 (basis)	
agree	65 (29.5)	25 (83.3)	11.92 (4.37–32.51)	< 0.001	12.38 (4.06–38.30)	< 0.001
The vaccine is safe						
not agree	130 (59.1)	6 (20.0)	1.00 (basis)		1.00 (basis)	
agree	90 (40.9)	24 (80.0)	5.78 (2.27–14.70)	< 0.001	1.99 (0.38–10.37)	0.528
Exposure at the workplace						
no	45 (20.5)	3 (10.0)	1.00 (basis)		1.00 (basis)	
yes	175 (79.5)	27 (90.0)	2.31 (0.67–7.97)	0.184	2.27 (0.43–11.92)	0.332

*cOR* crude odds ratio, *95% CI* 95% confidence interval, *aOR* adjusted odds ratio by all included variables in the multivariable model (Nagelkerke R^2^ = 0.516)

## Discussion

In this study, a specific risk group – healthcare workers - were vaccinated only at 12%. After several years of decline in the proportion of vaccinated inhabitants, only 3.3% of all Slovenian inhabitants were vaccinated in the season 2014/15, most in the age group over 65 years (11%) where the percentage of vaccinated patients was similar to that of the group of health workers in the Koroška region [17]. The reasons behind the decision to vaccinate are known to be complex. In this study they were mostly connected with the psychological determinants, such as perceived risk of disease, past experience with the vaccine and attitudes toward vaccination and vaccine. Contextual factors – free and accessible vaccine – were mentioned, too. Nurses were more negatively oriented toward vaccination than physicians.

Our study showed a low vaccination rate of primary healthcare professionals. The studies from other countries show a wide range of vaccination coverage of health professionals [9–11]. American data indicate a 40% rate of vaccination coverage of health professionals; their target is to reach 60% [19, 20]. Socan et al. showed that 41.7% of Slovenian physicians and dentists were vaccinated for seasonal and pandemic influenza in 2009/10 [12]. Among them, family physicians were vaccinated at 57.6%, while only 27% were vaccinated in this study. If we consider all nurses (practice nurses, registered nurses, community nurses and nurse carers) only 12 out of 129 (9%) were vaccinated, and if we include paramedics, who are registered nurses by their education but work in the emergency services and not in the family medicine team, it lowers to 8%. This could be another reflection of a well-known negative trend in vaccination coverage among the population in the last years [17].

The highest percentage of vaccinated health professionals in 2014/15 were those employed by private contractors (the team represents one physician and one practice nurse) (23.8%) and healthcare centres (18.5%), while the vaccination coverage was extremely low amongst employees of nursing homes (2.5%) and nil at the regional Healthcare emergency centre (0%). We were particularly surprised by the low vaccination coverage of nursing home health professionals, since their clients are highly endangered. It was much lower than the vaccination rate found in other studies in nursing homes [21]. We explain these results with the fact that the largest share of health workers in nursing homes are nurses and the vaccination coverage in this professional group is found to be low. Additionally, according to 2008 data, only 69% of nursing home residents were vaccinated, i.e. a third of them were not vaccinated and therefore were exposed to infection [22].

### Factors associated with the decision to vaccinate

In comparison to studies in other countries [9–11, 23, 24] where the most frequent reasons for vaccination were self-protection and free and easy access to vaccines, this study showed similar findings. The greatest motivator for vaccination of health professionals was the awareness that they are in the risk group for infection (83% of vaccinated respondents reported it). It was followed by the tendency for self-protection (70%), which was statistically not significantly behind, as it is essentially very similar and the protection of family members and co-workers (60%). Despite the fact that vaccination of health professionals is highly recommended, also for the indirect protection of patients, this was clearly a lesser factor (46.7%) in their choice to vaccinate.

In the bivariate analysis of factors associated with vaccination, the presence of a chronic illness, older age, being informed about influenza, belief in vaccine effectiveness and free vaccinations for employees were all significant, partly similar to Sočan et al., who showed that the factors associated with the decision to vaccinate are older age, being a hospital employee, being a vaccinator and having a chronic illness [12].

The multiple logistic regression model showed that the decision to vaccinate is influenced by age above 50 years, similar to other studies [25] and the respondents’ belief that the vaccination is effective. Interestingly, in our model, trust in professional recommendations and public health or lack of professional information to vaccinate was not important to our participants, although they were important factors for vaccination in the systematic review of Prematunge [26].

### Factors influencing the decision not to vaccinate against influenza

So-called hesitancy to vaccination [27] has been in the centre of research interest in the last years. This study shows that in the 2014/15 season, 220 health professionals (88.0%) were not vaccinated. The most common reasons against vaccination were listed in two groups: health professionals do not feel the need for vaccination (37.7%) and have a negative attitude toward vaccination, either from a first-hand bad experience (30.5%) or doubt in the vaccination’s effectiveness (37.3). Similar results were reported by foreign studies [9–11, 20]. This specific influenza hesitancy needs special investigation for further explanation. In the Schmid systematic review, lack of confidence in vaccine and vaccination was found to be the most important barrier for healthcare professionals [27].

Lack of information or insufficient awareness was mentioned by other studies as one of the most important reasons for refusing vaccination [9–11]. According to the respondents of this study, this did not impact the decision to vaccinate, as only 3.6% of health professionals mentioned it and statistically, it was significantly lagging behind the two primary reasons. In expert debates on mandatory vaccination for health professionals, however, it was emphasized that the decision depends on scientific evidence proving the effectiveness of the vaccine, its benefits, and its burdens and risks [28].

In this study, another important factor associated with the refusal to vaccinate turned out to be the respondents’ occupation. The multiple logistic regression model showed a negative association with the profession of nurse. Our study does not present an answer explaining the causes. In the literature, however, we found studies that specifically investigate answers to this issue. In Switzerland, a qualitative study was conducted among nurses from two different hospitals. The interviews showed that they value and maintain a healthy life-style. They do not believe that influenza vaccinations are beneficial; on the contrary, they consider them to be harmful (falling ill post-vaccination). They are afraid of the side effects and they have doubts in the vaccine’s effectiveness. Amongst their reasons not to vaccinate was also the wish for autonomous decision-making about one’s body and health, and distrust in the environment and scientific study findings [29].

### Limitations of the study

All primary health professionals of the Koroška region were invited to participate in the study, but we could only include the institutions and private contractors that agreed to participate. Hence, the sample was not representative. This affects the generalizability of the results although the response rate among the participants was good (74.8%). The limitation of the study was also the fact that it focused on a single region in Slovenia, which is why the subgroups of health professionals were small and the generalization of the results for the entire population of health professionals in Slovenia is not possible. It was also not possible to analyse motivators and barriers according to the specific professional groups or to find any differences among them. Further on, the cross sectional design of the study did not allow any causal relationship to be established between the variables. Finally, as to our knowledge there are no validated questionnaires on this topic, the questionnaire we used was designed according to the literature review, aims of the study and previous qualitative data which limits the comparison of factors associated with influenza vaccination to other studies.

## Conclusions

The downward trend of influenza vaccination amongst health professionals continued in 2014/15. The reasons for low vaccination in health professional group are complex. Health professionals need to be more aware that it is their professional duty to protect patients against influenza infection spread by healthcare workers. This is especially important for some professional groups, while physicians and dentists seem to be aware of it.

The vaccination of health workers is especially important for the protection of the patients in nursing homes. Further research to prove this effect and qualitative type research should be performed with some health professional groups, such as nurses, to get an insight in any specific aspects and possibilities for the improvement of influenza vaccination coverage.

## Abbreviations

NIJZ: National Institute of Public Health
WHO: World Health Organization
